# Fever of Unknown Origin: A Rare Diagnosis Requiring High Suspicion

**DOI:** 10.7759/cureus.75330

**Published:** 2024-12-08

**Authors:** Beatriz R Marques, Filipa Seixas, Mariana Nunes, Sofia L Costa, Victor Paz

**Affiliations:** 1 Internal Medicine, Unidade Local de Saúde de Trás-os-Montes e Alto Douro, Chaves, PRT; 2 Hematology, Unidade Local de Saúde Trás-os-Montes e Alto Douro, Vila Real, PRT; 3 Pathology, Unidade Local de Saúde Trás-os-Montes e Alto Douro, Vila Real, PRT

**Keywords:** fever of unknown origin, hemophagocytic lymphohistiocytosis, hemophagocytic syndrome, hyperferritinemia, undiagnosed fever

## Abstract

Fever is a classic reason for hospital visits, sometimes requiring admission. Its etiologies are numerous, ranging from simple and relatively common conditions to rare and complex pathologies, for which the differential diagnosis can present a true challenge for internists. A 78-year-old healthy female is referred to the emergency department due to marked fatigue for the past four months, with no other symptoms. Physical examination revealed fever, significant lower limb edema, and low blood pressure. Laboratory results revealed anemia and thrombocytopenia, associated with high ferritin and lactate dehydrogenase levels (1924 U/L and 1519 U/L, respectively) and mild hyponatremia (133 mEq/L). A thoracic-abdominal-pelvic CT scan showed only a splenomegaly of 17 cm without other significant findings. No microorganisms were found in multiple cultural samples, and fever persisted despite two courses of antibiotics. Viral serologies and zoonosis panel were negative. A bone marrow study was conducted to help explain the cytopenias, which revealed hemophagocytic cells, confirming the diagnosis of hemophagocytic syndrome. She started with systemic corticosteroid therapy, which improved her symptoms, and a few days later, it was confirmed a diffuse large B-cell lymphoma was the etiology. Because of its mostly unspecific manifestations, hemophagocytic syndrome requires a high degree of suspicion for timely diagnosis and treatment.

## Introduction

Fever has preoccupied physicians since the earliest days of clinical medicine. Fever is a classic reason for hospital visits, has been scrutinized in recent decades, and remains a major diagnostic challenge. Its etiologies are numerous, ranging from simple and relatively common conditions to rare and complex pathologies with very different prognostic outcomes, for which the differential diagnosis can present a true challenge for internists [1]. A fever of unknown origin is defined as a temperature >38.3˚C recorded several times for >3 weeks despite appropriate initial inpatient or outpatient evaluation [2]. Constant re-evaluation of clinical data is essential considering the dynamic changes in disease patterns.

The authors present a clinical case of a prolonged fever subjected to extended investigation, leading to a diagnosis of hemophagocytic lymphohistiocytosis (HLH) caused by lymphoma. This syndrome is a rare condition characterized by excessive immune system activation. It more commonly affects children but can occur at any age [3]. This case report aims to alert other physicians to this rare disease, which can be potentially fatal if not treated in time.

## Case presentation

A 78-year-old female, a former tailor with a known medical history of hypertension, dyslipidemia, and vertiginous syndrome, presents to the emergency department due to marked fatigue for the past four months. No respiratory, cardiac, genitourinary, or neurological symptoms were reported. The patient lived in an urban environment with potable water, denied consuming raw milk or raw milk products, and had no contact with animals. On examination, she presented with fever, significant lower limb edema, and arterial pressure of 96/55 mmHg without other noticeable findings. Laboratory results revealed severe microcytic anemia, thrombocytopenia, elevated lactate dehydrogenase (LDH), no hepatic cytolysis, and normal renal function. The patient reported no blood losses. She was admitted to the internal medicine ward for further investigation. The initial blood findings are shown in Table 1.

**Table 1:** Analytic study at admission aPTT: Activated partial thromboplastin time; ALT: Alanine transaminase; AST: Aspartate transaminase; INR: International normalised ratio; MCV: Mean corpuscular volume; γ-GT: Gamma-glutamyl transferase

Parameter	Result	Reference Value
Hemoglobin	7.3	12-16 g/dL
MCV	78.0	87-103 fL
Leucocyte count	6790	4-11 x 10^3^/uL
Platelets	94000	150-400 x 10^3^/uL
Urea/creatinine	40/1.0	<50/0.5-0.9 mg/dL
Sodium/Potassium	133/3.9	135-147/3.7-5.1 mEq/L
AST	38	<35 U/L
ALT	17	<33 U/L
γ-GT	9	7-32 U/L
Alkaline Phosphatase	46	35-105 U/L
Total Bilirubin	0.5	<1.2 mg/dL
Lactate Dehydrogenase	1519	135-214 U/L
INR	1.4	<1.2
aPTT	31.5	27-38 seg
C Reactive Protein	6.32	<0.5 mg/dL

**Figure 1:**
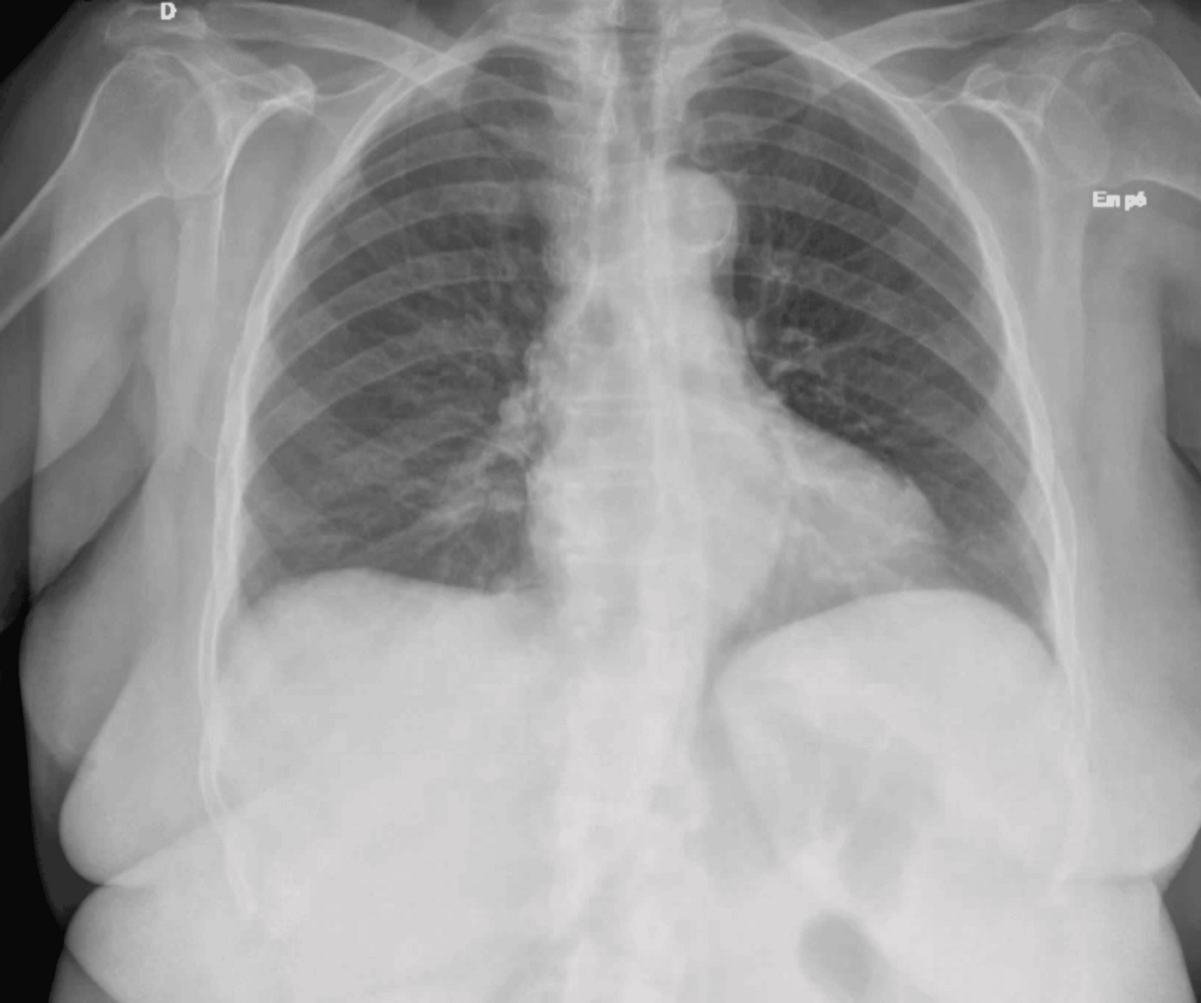
Chest radiography at admission

**Figure 2:**
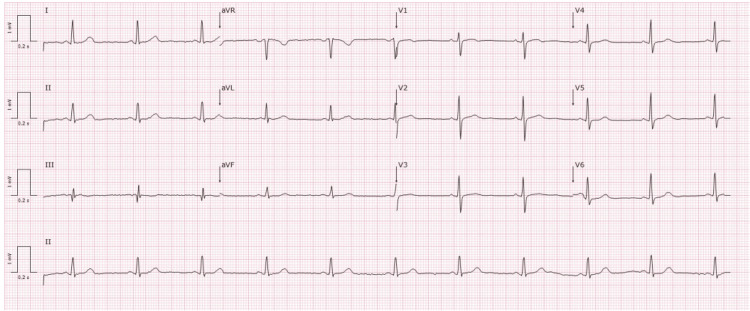
Electrocardiogram at admission

**Figure 3:**
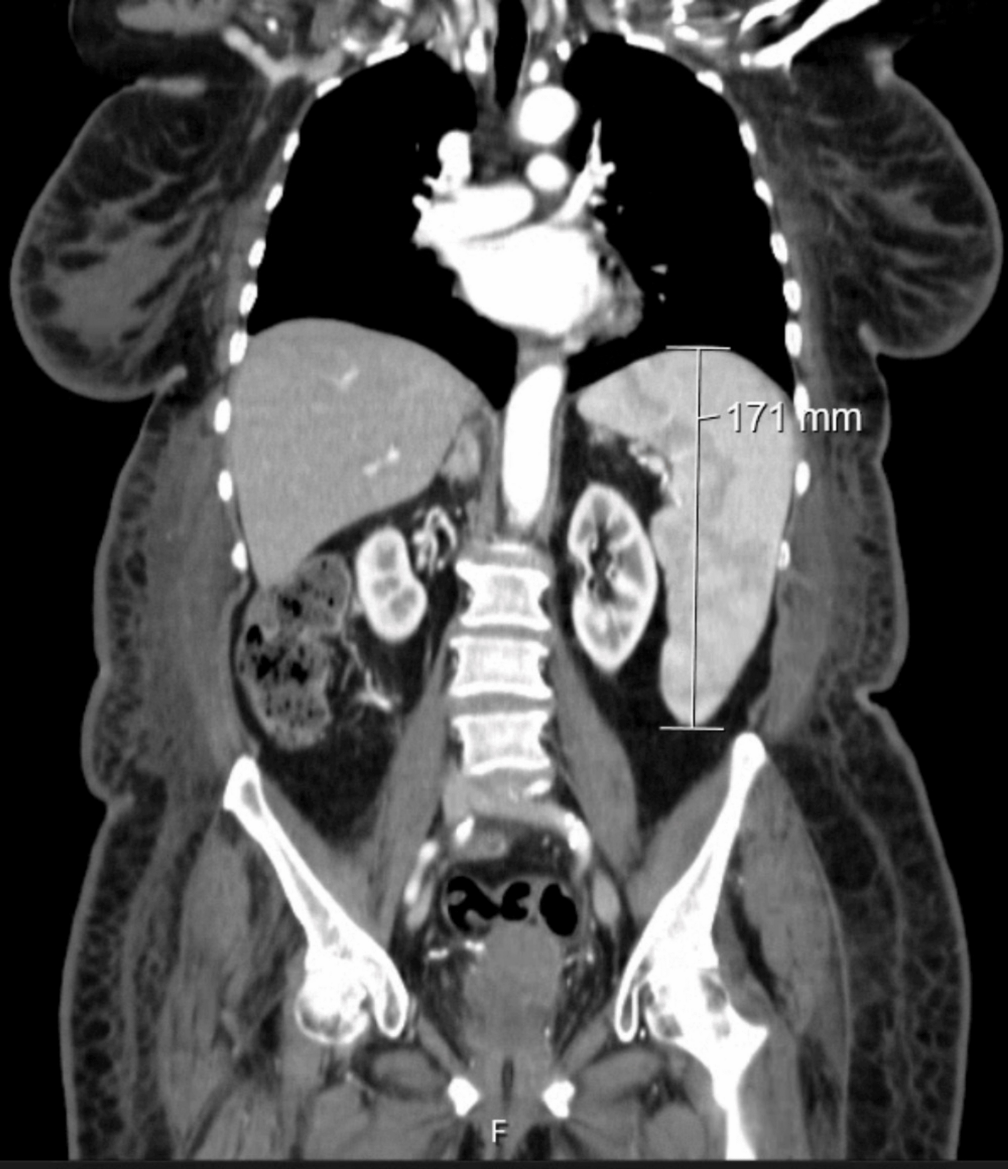
Computed tomography showing an enlarged spleen

The chest radiography was normal (Figure 1), as well as the electrocardiogram (Figure 2). A full-body computed tomography (CT) was performed, showing mild hepatic steatosis and an enlarged spleen (17.1 cm) without any other features worth mentioning (Figure 3).

**Figure 4:**
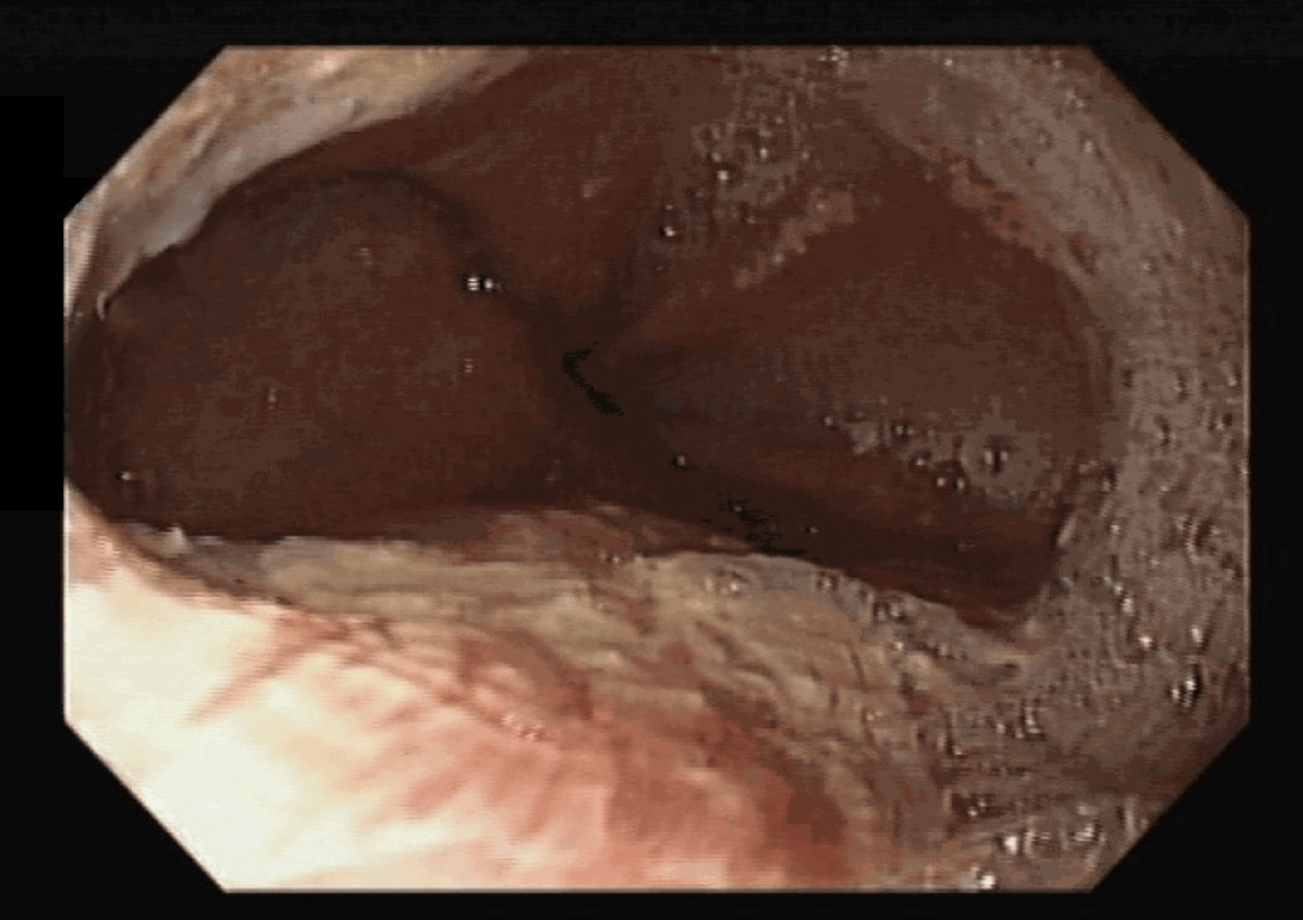
Severe reflux esophagitis (grade D)

She was submitted to an upper digestive endoscopy, which revealed severe reflux esophagitis (grade D) affecting 75% of the esophagus circumference (Figure 4). The colonoscopy did not show any suspicious lesions.

An extended analytic study was performed to understand the cause of anemia, thrombocytopenia, and constitutional syndrome (Table 2). Whenever necessary, the patient was transfused with erythrocytes.

**Table 2:** Extended analytic study Anti-dsDNA: Anti-double stranded deoxyribonucleic acid; ANA: Antinuclear antibodies; ANCA: Antineutrophil cytoplasmic antibodies; Anti-CCP: Anti-cyclic citrullinated peptide; Anti-PR3: Anti-proteinase 3; ESR: Erythrocyte sedimentation rate; Ig: Immunoglobulin;  TIBC: Total iron binding capacity; TSH: Thyroid-stimulating hormone; T4: Thyroxine

Parameter	Result	Reference Value
Reticulocytes	0.105	0.025-0.090 x 10^12^
Blood smear	Poikilocytosis	-
Serum Iron	18	37-145 ug/mL
TIBC	160	228-360 mg/dL
Transferrin saturation	10.1	>20%
Serum Ferritin	1924	15-150 ng/mL
Folic acid	2.9	2.2-17.5 ng/mL
Vitamin B12	387.9	191-663 pg/mL
Haptoglobin	154	30-200 mg/dL
TSH	1.89	0.27-4.2 mIU/L
T4	7.72	8.2-21 pmol/L
Serum Albumin	2.9	3.4-4.8 g/dL
Serum Total Proteins	5.2	6.6-8.7 g/dL
Protein electrophoresis	Normal	-
IgG/IgM/IgA	All normal	-
24h urine Proteins	159	<150 mg/24h
Uric acid	5.8	2.3-5.7 mg/dL
ESR 1ª hour	65	0-30 mm/1ªh
Fibrinogen	406	200-400 mg/dL
Anti-dsDNA antibodies	<1:10	<1:10
ANCA’s	<1:20	<1:20
ANA’s	Negative	-
Anti-phospholipid antibodies	Negative	-
Anti-CCP antibody	Negative	-
Anti-PR3 antibodies	Negative	-
Complement proteins	100/14	90-180/12-36 mg/dL
Serum Triglycerides	320	<150 mg/dL

The patient was started on empiric antibiotics with ceftriaxone, and several exams were conducted to find the source of the fever. The transthoracic echocardiogram did not show signs of endocarditis. Table 3 shows the microbiology and serology results.

**Table 3:** Analytic results in search of an infectious agent :CMV: Cytomegalovirus; EBV: Epstein-Barr virus; HBV: Hepatitis B virus; HCV: Hepatitis C virus; HIV: Human immunodeficient virus; Ig: Immunoglobulin

Parameter	Result	Parameter	Result
Blood cultures (2^nd^ day)	Negative	CMV antibodies IgG/IgM	Positive/Negative
Blood cultures (8^th^ day)	Negative	EBV antibodies IgG/IgM	Positive/Negative
Blood cultures (17^th^ day)	Negative	*Chlamydia trachomatis* IgG/IgM	Negative/Negative
Urine sediment	Normal	Wright Reaction	Negative
Urine culture	Negative	Rose Bengal	Negative
Respiratory virus panel	Negative	*Coxiella Burnetti* antibodies IgG/IgM	Positive/Negative
HBV antibodies	Non-reactive	*Mycoplasma pneumoniae* antibodies IgG/IgM	Negative/Negative
HBV HBs antigen	Non-reactive	*Ricketsia spp* antibodies IgG/IgM	Negative/Negative
HCV antibody	Non-reactive	*Parvovirus* antibodies IgG/IgM	Negative/Negative
HIV antibody	Non-reactive	Zoonoses Panel	Negative

**Figure 5:**
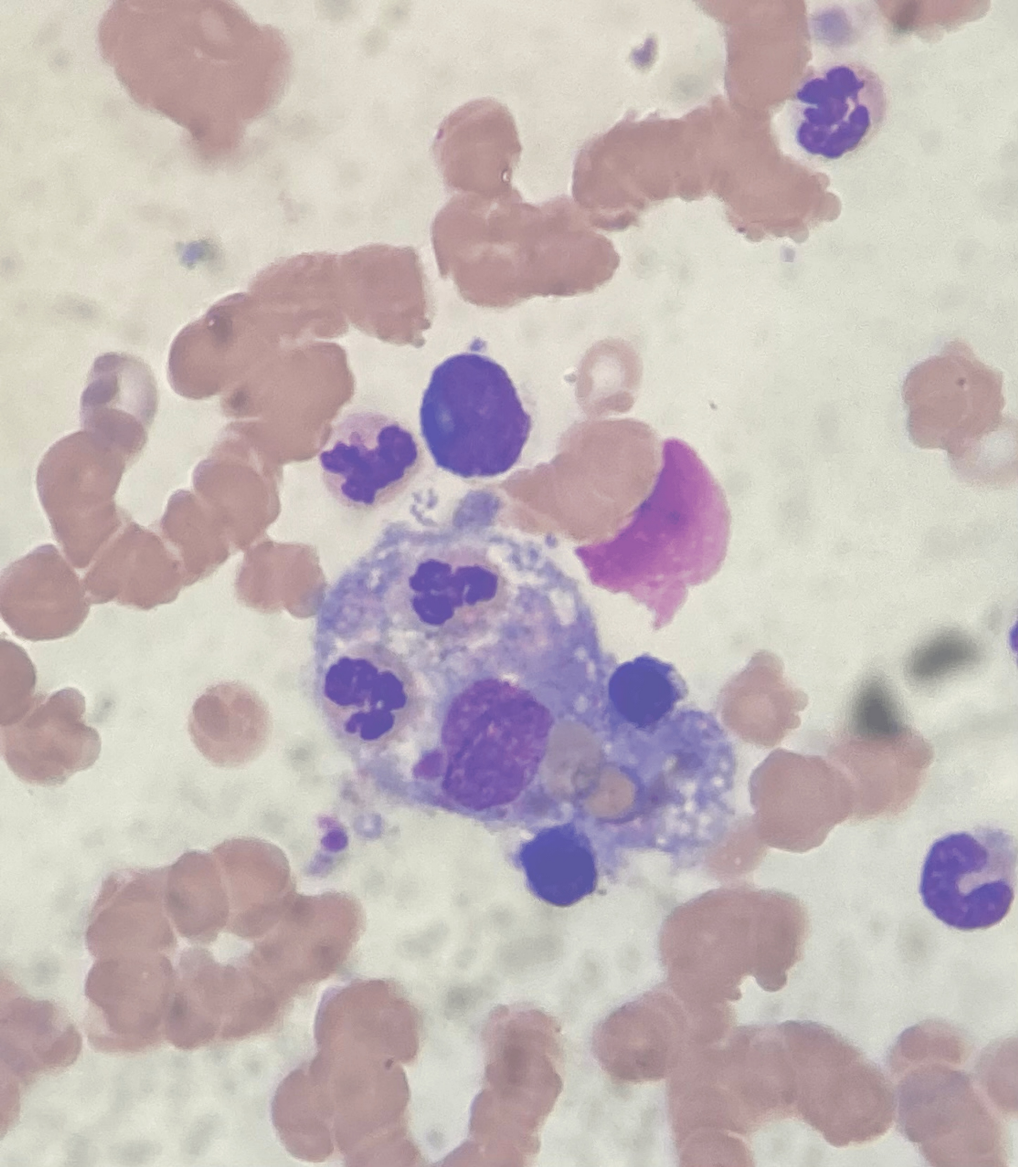
Macrophage phagocyting cellular elements

The fever persisted after three weeks of exams, two cycles of antibiotics, and no microbiologic isolate. High ferritin levels associated with fever, anemia, and thrombocytopenia, with no other explanation, started to raise suspicions of hemophagocytic syndrome, so serum fibrinogen and serum triglycerides were measured (Table 2). Finally, on the 20th day, a bone marrow study (with bone marrow biopsy) revealed hemophagocytic cells (Figure 5), confirming the diagnosis of hemophagocytic syndrome, and systemic corticosteroid therapy was initiated.

**Figure 6:**
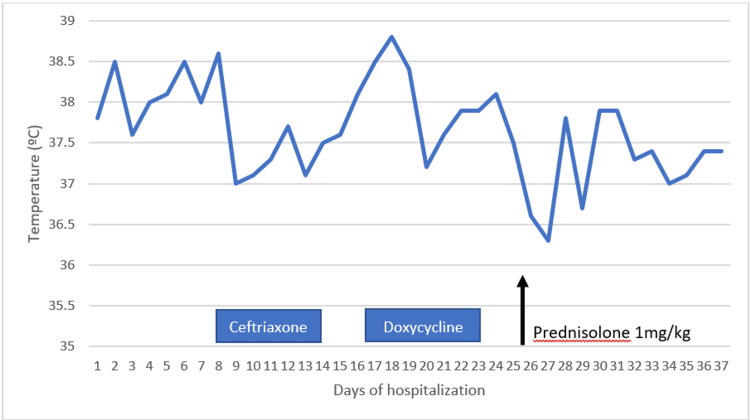
Temperature variation through hospitalization

**Figure 7:**
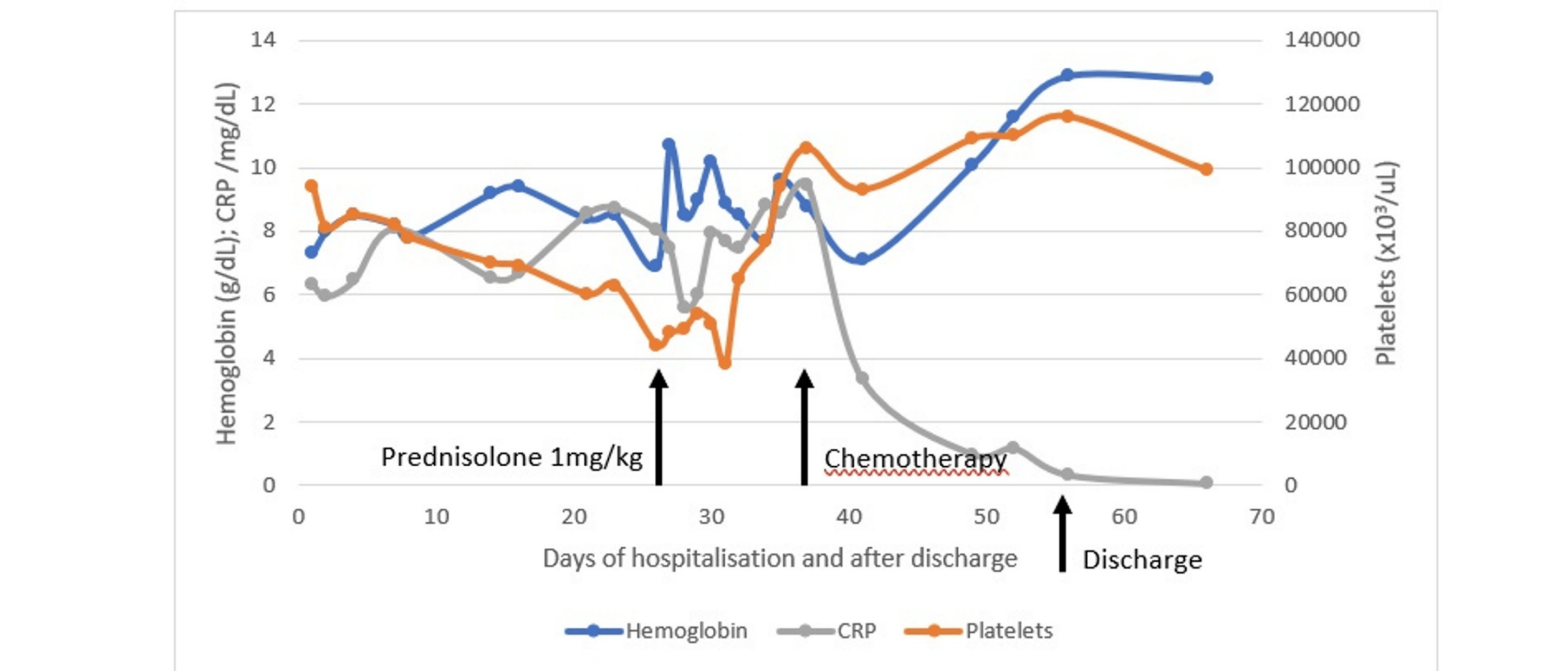
Hemoglobin, platelets and CRP variation through the hospital stay and after discharge CRP – C Reactive Protein

This confirmed the diagnosis, fulfilling 6 out of 8 criteria of the revised diagnostic guidelines for HLH-2004 [4]. Figures 6-7 show the evolution of the hyperthermia and some laboratory parameters, respectively, during four weeks of hospitalization.

During the hospital stay, the patient suffered a clinical worsening, with a transitory need for vasopressor support due to aggravated hypotension. After receiving the results from bone marrow immunophenotyping, a diagnosis of diffuse large B-cell lymphoma was confirmed, and targeted chemotherapy was initiated the following day with slow and steady improvement. Figure 7 illustrates the evolution of some analytic parameters through the hospital stay and the impact of different treatment phases.

## Discussion

Hemophagocytic syndrome, or hemophagocytic lymphohistiocytosis, is characterized by an excessive activation of the immune system, more commonly affecting children but occurring at any age. It is classically divided into two groups - primary and secondary. Primary HLH occurs in children, typically within the first year of life, and is due to recessive genetic mutations that affect the function of T-NK lymphocytes and cytotoxic T-cells. In adults, secondary HLH arises from an underlying predisposing condition that disrupts immune regulation, such as neoplasms (particularly hematological), autoimmune diseases, or immunodeficiencies, and/or is triggered by bacterial, viral, fungal, or parasitic infections [5,6]. In a French study involving 162 adult patients, the most commonly associated condition with HLH was neoplasia (60%), followed by infection (25%) and autoimmune diseases (3%) [7].

Although mostly described in immunocompromised hosts associated with viral infections [8], most patients with HLH are not obviously immunosuppressed. When associated with a neoplasm (malignancy-associated hemophagocytic syndrome), it may either be the presenting clinical picture and initially mask the underlying malignancy or develop during the treatment for a known malignancy [4].

Clinically, HLH manifests as a fever associated with progressive dysfunction across multiple organs. The initial presentation can vary, often resembling common infections, hepatitis, or encephalitis, and may sometimes be diagnosed as a fever of unknown origin [9]. For these reasons, HLH is most known for its major diagnostic and therapeutic difficulties [4].

The first diagnostic guidelines were published by The Histiocyte Society in 1991 [10], which were later updated in 1994 with five criteria for its diagnosis (HLH-94 diagnostic criteria). In 2007, three more components were added [4] for a total of eight conditions (HLH 2004 criteria): fever, cytopenias involving two or more cell lines, hypertriglyceridemia or hypofibrinogenemia, splenomegaly, hemophagocytosis seen in bone marrow, lymph node or spleen, absent or low NK cell activity, raised serum ferritin, elevated sIL2R. HScore is another criterion used in adults to diagnose HLH [11].

The treatment involves, on the one hand, the removal of the trigger for HLH, whether it is infectious, neoplastic, or autoimmune, and, on the other hand, the management of the dysregulated immune system. Systemic corticosteroids are consensually the first-line therapy, with additional use of protocols based either on etoposide (as recommended by HLH-2004) or CHOP (cyclophosphamide, doxorubicin, vincristine, and prednisone) when cytotoxic drugs are necessary, such as the presented case. However, no consensus exists regarding the optimal initial therapy or its duration [5].

## Conclusions

HLH is a rare disease that was fatal during the initial years when no proper diagnostic or treatment protocols were available. The presented case aims to illustrate a rare but real and likely underdiagnosed condition. Due to the nonspecific signs and symptoms, combined with a poor prognosis if left untreated, HLH requires a very high index of suspicion for a timely diagnosis and treatment.
